# The impact of music-imaginative pain treatment (MIPT) on psychophysical affect regulation – A single case study

**DOI:** 10.3389/fpain.2022.943890

**Published:** 2022-09-22

**Authors:** Sina Glomb, Irina Böckelmann, Jörg Frommer, Susanne Metzner

**Affiliations:** ^1^ Department of Psychosomatic Medicine and Psychotherapy, University Hospital Magdeburg, Otto-von-Guericke-University Magdeburg, Germany; ^2^ Department of Occupational Medicine, Medical Faculty, Otto-von-Guericke-Universität Magdeburg, Germany; ^3^ Faculty of Philosophy and Social Sciences, Augsburg University, Germany; ^4^ Faculty of Medicine, Augsburg University, Germany

**Keywords:** somatoform pain disorder, music-imaginative paint treatment, affect regulation, trauma, heart rate variabiity

## Abstract

Music-imaginative Pain Treatment (MIPT) is part of the multi-professional treatment plan for hospitalised patients in departments for psychosomatic medicine. MIPT is an intervention that encourages the patient to create music representing pain and relief from pain and promotes active engagement and self-reflection. This single case study of a 46-year-old female patient diagnosed with chronic pain disorder with somatic and psychological factors includes narrative, demographic, psychometric, and cardiophysiological data. During the interventions, early childhood stress, which is a risk factor for developing chronic pain, turns out to be a crucial focal point in therapy and conspicuous in her handling of the music. Social trauma is considered an appropriate concept for a deeper understanding of the case.

## Introduction

How do pain perception and vegetative regulation of the cardiophysiological system (heart rate variability; HRV) change through music-imaginative pain treatment (MIPT) in a chronic pain patient? What is the development and sound of self-composed music for pain and relief? What influence do events from one's biography have on the course of treatment? How does the patient develop an awareness of the impact of biographical events on chronic pain during MIPT?

These questions are central in the single case study of Mrs S., who participated in a clinical study by Metzner et al. (2022) (1) with patients suffering from a chronic pain disorder and undergoing psychosomatic inpatient treatment. Rather than presenting outcomes this multi-perspective analysis aims to gain a differentiated insight into the complex impact levels, and their interconnections, that come into effect in MIPT. This case study presents the chronology of the treatment process. It successively integrates the surrounding quali-quantitative data, including psychometric data on (a) the baseline assessment at the beginning of the treatment, (b) the subjective pain intensity (pre-post), and (c) cardiophysiological data collected through Electrocardiogramm (ECG) and subsequent HRV analysis. Additionally, (d) narrative and musical data are collected through audio recordings.

## Context, setting, intervention

In the Department of Psychosomatic Medicine and Psychotherapy at the University Hospital Magdeburg all kinds of psychosomatic disorders were treated. The multi-professional, complex treatment was based on a psychodynamic model. The treatment typically lasted 12 weeks. During this period, the patients received group and individual psychotherapy as well as music-, art-, social- and movement-therapy. Patients with a chronic pain disorder were offered MIPT during the first weeks of their inpatient stay, encompassing 4 sessions within 2 weeks.

### Music-imaginative pain treatment (MIPT)

Music-imaginative pain treatment (MIPT) is an intervention that was initially developed as a form of “entrainment” within a single session (2, 3); later, it was established as a manualised treatment (4). Its professional implementation requires special further training.

MIPT takes place in an individual setting in a room equipped with a great variety of musical instruments. It comprises four treatment phases (I–IV), with three to four 50 min-sessions: firstly, the pain experience is explored in a detailed narrative interview and quantitatively recorded using a visual analogue scale (VAS) (see Section “Cardio-physiological data”) (I). In the second step, the patient creates two sound compositions with the assistance of the therapist to express the pain experience (Pain Music; PM) and the idea of pain relief (Healing Music; HM) (II). No previous musical training is required for this. In the application phase (III), the music therapist plays the two compositions to the patient. The patient indicates the start and stop of PM and of HM, and controls the sequence of musical events as well as the music's tempo and dynamics. Finally (IV), the patient's experience is reflected on in a conversation, and new insights for the further course of therapy are discussed in the department. The role of the therapist is to provide a supportive framework for the exploration of the pain's characteristics, to assist the patient in the musical realisation of the pain experience and the ideas of relief, to empathise with the patient's experience both emotionally and musically, and to support the patient in this challenging, sometimes confrontational work. Clinical experience shows that MIPT gives the patient space to gain more flexibility when dealing with the pain experience and the affective attitudes toward pain more flexible. It increases the feeling of self-efficacy and promotes communication skills (5). In some cases, there is an insight into the underlying bio-psycho-social conditioning structure of chronic pain (6).

## Case study: Mrs S.

### Medical history, diagnoses and psychopathological findings at admission

#### Medical history

Mrs S. had been on sick leave for more than two years before admission. She had already been treated several times as an inpatient and day-care psychosomatic patient and once as a psychiatric patient due to a suicide attempt eight years ago. The focus so far had been a panic disorder, for which she was no longer able to leave the house. Mrs S. had been suffering from anxiety attacks for 20 years, for the last five years increasingly connected to problems in her marriage and at work. Due to the severe pain, especially in her legs, a rheumatological analysis was carried out, and fibromyalgia was diagnosed.

#### Diagnoses and psychopathological findings

• Chronic pain disorder, ICD 10: F 45.4 L through intervertebral disc protrusion L5/S1 on the right side
• Panic disorder, ICD-10 F41
• Combined personality disorder with dependent and histrionic components, ICD-l0 F61

The Symptom Checklist 90 (SCL 90) (7), which measures the current, perceived impairment due to physical and psychological symptoms, was used to assess the symptom burden on admission to the clinic (time: T0). Mrs S.'s basic psychological distress, according to the global characteristic value GSI (Global Severity Index), was in the range of “significantly increased” with 69 points. In comparison, the subscale “Obsessiveness” (72 points) was strongly increased, and the subscale “Phobic Anxiety” (77 points) was very strongly increased.

In addition, the short version of the Childhood Trauma Questionnaire (CTQ) (8) was used to assess emotional, physical and sexual abuse as well as emotional and physical neglect in childhood. In the case of Mrs S., the measured scores for three subscales resulted in the classification as “severe”: “emotional abuse” (17 points), “emotional neglect” (17 points), and “physical neglect” (10 points).

### Biography and current living situation

Mrs S. was born and grew up in a small town in East Germany during the time of the German Democratic Republic (GDR) as the second child of state scientists and Socialist Unity Party (SED) members. Her older brother was favoured, while the patient only received recognition when she was in the newspaper for extraordinary achievements. If she got average grades at school, she was immediately grounded. If she was not on the podium in sports, she was criticised for not trying hard enough. Often she had to do additional training in the evening. Furthermore, she had to help a lot with household chores. If anything did not add up to the patent's standards, she was beaten by her mother with her “slipper” or by her father with his hand. As a teenager, her parents got her involved in politics, and she became a SED member herself.

Her first profession as a cook she did not choose herself. After two decades, she requalified to become an office clerk.

When Mrs S. got pregnant at the age of 20, she married the child's father against her parents' will. After the opening of the inner-German border, they moved to West Germany with their daughter. She felt oppressed in this marriage, that later turned violent and she decided to divorce her husband after 18 years of marriage. Then, she moved back to East Germany, where she married a second time. Mrs S. has been concerned about her husband's chronic illnesses.

At present Mrs S. is employed full-time in a zoo and pet shop but has been unable to work for 24 weeks. She states that she often worked there six days a week, for up to 12 h. This commitment at work was unnecessary and led to conflicts with her colleagues. She also went to the gym four times a week and worked out until completely exhausted.

### Mrs S. in music therapy^1^

Mrs S.'s MIPT sessions were audio-recorded. The following account summarises her therapeutic process and integrates literal quotations from the patient.

#### Interview on pain (MIPT phase I)

Mrs S. describes her pain as starting from the hip and going through the legs into the feet and toes. Her arms were sensitive to pressure. The quality of the pain in the legs was tearing, tingling, like “stinging nettles”. Mrs S. reported that she had been suffering from this pain continuously for two years. It is particularly severe in episodes where she suffers from feelings of weakness and gait disturbance. There is no apparent trigger. From her perspective, the pain comes from work overload, stress, and anger in her private life: “As if the soul says to the body: ‘She does not listen to me; you have to tell her’.” Tears come to her eyes.

Overall, Mrs S. feels exhausted and depressed. She observes an intense need for sleep and increasing forgetfulness. She is afraid of being considered a malingerer because all the treatment attempts with analgesics have had no effect. She had always had back pain in the past, but now she feels anger, despair, and helplessness. She sometimes seeks to hurt herself by trying to defy the pain actively, presses her toes firmly on the ground, walks barefoot on stones, and pinches her calves until they bruise: “There, I’ve shown you.”

#### The composition of pain and healing music (MIPT phase II)

At the beginning of the session, the patient emphasises that her legs are always cramped and tense. When asked how she imagines music that describes her experience of pain, she answers: “Loud, bright, shrill. Like screaming.” In the room equipped with numerous musical instruments, Mrs S. and her therapist search for suitable sound qualities. They try out various instruments until the patient decides on the marimba. A single bar, the *D*″, is struck with the wooden mallet quickly, loudly and in a constant penetrating pulse. This sound corresponds to the sharp, poignant quality of her pain. Then the next instrument is the cello. The strings are struck with the bow directly behind the bridge to express the loud, shrill scream that sounds “like a circular saw”. The third instrument Mrs S. chooses is the bass slit drum. A specific note is struck quickly and consistently with a soft woollen mallet. The effect is a droning sound that the patient associates with her depressed state and her racing heart during a panic attack.

The patient is visibly tense as she selects and listens to the composition of sound qualities. “This really is it,” she says. She wrings her hands and mentions immediate physical reactions (cramping in the legs, feeling hot, sweaty hands, racing heart). She says: “I am amazed that the sounds have such an effect on me. Without me having to do anything. It is automatic.” The therapist and patient exchange ideas about how this music sounds rigid, mechanical and inhuman, like being in a factory with machines punching out metal. Mrs S. says: “If only I could sit down somewhere and let my feelings out. If only I could cry, but I can’t.”

The work on the pain music has required the whole session. Therefore, the healing music piece is being composed in another session. The patient is tense when she arrives at this session. She says that there had been an altercation on the ward. Nevertheless, she gets involved in the MIPT. She takes up a previously expressed idea about the composition of soothing sounds: they should be free, playful and reminiscent of a holiday at the Baltic Sea with her husband and dog. When the therapist asks her about childhood memories of the Baltic Sea, her mood changes. Even today, she feels disgust and shame when she thinks about having been forced by her parents to go to the nudist beach. Before those family holidays, she was often aggressive to other children or sick.

Musically, Mrs S. starts with the ocean drum^2^. She explores herself, sees herself in the water, in a playful fight with the waves, wants to be like the water, powerful, unstoppable, alive, “then nobody can harm me”. Her family (parents, brother), on the other hand, would be intimidating “opponents”. This fight has two sides: liberation from confinement and paternalism, but also pressure and physical exertion. However, she rejects the first way out of the situation: three soft sounds on the sansula^3^. Instead, she chooses the children's harp. The name alone seems to appeal to the patient. The lower 5–6 strings are to be arpeggiated irregularly, randomly, and with some louder notes in between. Nevertheless, the music seems a little mechanical.

Mrs S. determines that the the healing music shall begin with the children's harp, and then the ocean drum is added. The patient remarks that she has no cramped legs and feet and no sweaty hands at the moment.

#### Application of music (MIPT phase III)

During the MIPT session, the 24 h-ECG is running (see Section “Cardio-physiological data”). The device was put on beforehand so that the patient could get used to it before music therapy. The patient is excited and curious about what to expect. She has already felt anxiety on the way to the music therapy room. The therapist explains the course of the session. Due to the ECG-measurement there is a 5-minute resting phase before the start of the pain music. The pain music lasts two and a half minutes. The stroking on the cello does not work as arranged; the sound is not shrill enough but rather rough and uneven. The patient demanded the booming pulse on the bass slit drum twice more intensely. The healing music could not be realised as planned. The playing on the children's harp became too lively, and the ocean drum's sound seemed too halting. The music ends after a total of 7 min and 33 s.

In the following conversation, Mrs S. expresses her exhaustion. The silence of the 5-minute resting phase has been experienced as torturous by her. It reminded her of her mother ignoring her for days as punishment. (The therapist would have skipped the quiet phase if Mrs S. had mentioned these memories before.) The end of the pain music she experienced as a degrading “begging for mercy”, also like in childhood. The pain intensity had risen to point 9 on the 10-point VAS. The therapist inquires about her reason for increasing the droning of the bass slit drum. “I wanted to push the pain to the extreme and prove how much I can take”. When the therapist confronts the patient with the fact that this could be seen as auto-aggression, connections between the childhood traumas and the chronic pain become clear. The patient is frightened and sad: “Because what I do is never good enough. I have always had to endure the punishments in my life.”

“The healing music had slowly brought relaxation, and the pain intensity decreased – in everyday life as well …” says the patient. But she can only endure moments of well-being for a short time because this triggers feelings of guilt and worthlessness. To avoid this she escapes again into task fulfilment and performance.

The patient leaves the session emotionally agitated, but at the same time, she seems more relaxed. She expresses relief that she has put it all behind her and needs to let it sink in.

#### Reflection (MIPT phase IV)

Mrs S. says that the last MIPT session had a strong impact. She had talked about it in other therapies and also with her husband. For her, it is so frightening that not only did her childhood experiences have such a significant influence on her, but also she treated herself just as badly for a long time. The patient, who previously had problems identifying and verbalizing her emotions recognizes now a conglomerate of bewilderment, sadness, bitterness and disappointment, but at the same time also longing, especially for her parents, her daughter and her granddaughter, with whom she has had no contact at all for a year. “The fact that I talked about my parents like that and couldn’t show any emotions kept me on edge. And my body then reacted in such a violent way – also to the music.”

Although the process has not been easy, Mrs S. experiences MIPT as a constructive process. The therapists offers the audio recording of the music for her own use or as a keepsake. Mrs S. likes to receive it, even though she may not listen to it, saying: “After all, it belongs to me – despite everything”.

## Quantitative data

### Pain intensity/10-step visual analogue scale (VAS) before and after

Pain intensity was measured at two points in time. In Table 1, both assessments (current and retrospective/forward-looking) are included.

**Table 1:** Self-assessment of pain intensity at different points in time (10-step visual analogue scale VAS).

Time/time span	Pain intensity
Current: Start of MIPT	6
Retrospective average over the last four weeks	8
End of treatment	4
Retrospective application phase (pain music)	9
Retrospective application phase (healing music)	6
Current: Completion of MIPT	4

### Cardio-physiological data

The HRV analysis of the application of self-composed music during MIPT as well as of entire day was included in analysis. The patient had a 24 h-ECG recording with the medilog R AR12 PLUS device (SCHILLER, Baar, Switzerland). That is a 3-channel ECG device with automatic detection of R waves, for which the sampling rate has been set to 1,000 Hz as recommended by the national guideline (9). Artefacts were manually removed from the raw ECG data using the medilog R DARWIN2 Enterprise processing software (SCHILLER, Baar, Switzerland) and prepared as Riva Rocci data (RR) interval series for HRV analysis. The program Kubios HRV Version 2.0, University of Eastern Finland, Kuopio, Finland (10, 11) was used to analyse the HRV parameters. The HRV parameters were determined in the time, frequency and phase domain.

#### Analysis of the 24-hour ECG recording

**Figure 1:**
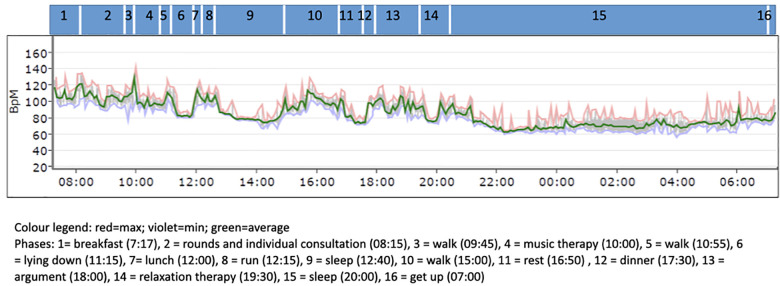
Course of heart rate (HR) (note: the time on the *X*-axis is the recording time from the time the ECG device was applied).

The assessment of the ECG recording over 24 h showed that the patient has a continuous still normal-frequency sinus rhythm with isolated ventricular extrasystoles with compensatory pauses (*n* = 30; 0.02%) and isolated supraventricular extrasystoles (*n* = 2). There are no higher grade volleys, and no prolonged pauses are seen. The mean heart rate (HR) was high, indicating higher sympathetic activation with 95 beats/min during the day and 73 beats/min during the night (reference values for females 45–46 years according to Lohninger, 2017, *p*. 232–33 (12): mean heart rate 24 h 69.7–78.1/Median 73.9 beats/min; mean heart rate day 75.57–84.4/Median 80 beats/min; mean heart rate night 61.1–69.3/Median 62.2 beats/min). Dynamic A (difference mean HR day/sleep) is strong for the 46-year-old, indicating, among other things, sustained activation during the day (reference values for females 45–46 years according to Lohninger, 2017, *p*. 234 (12): dynamic A 12.8–18.7/Median 15.7 beats/min). Over the entire recording period (23:59:59), this value was 87 beats/min and thus significantly above the average value for women of this age. The maximum HR was recorded at 09:59:52 at 144 beats/min (BMP) (see Figure 1) during the first minutes of the music therapy phase. The minimum HR was measured at 54 beats/min at 03:59:22 (during sleep).

Overall, the 24 h-ECG recording is to be considered within the normal range.

#### Evaluation of HR variability from the 24 h-ECG

**Figure 2:**
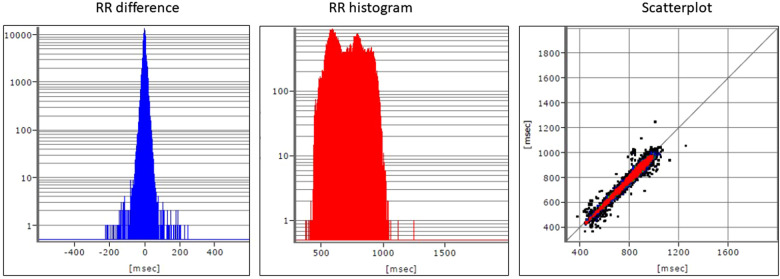
HRV parameters from the time domain as well as the representation of the scatterplot.

**Figure 3:**
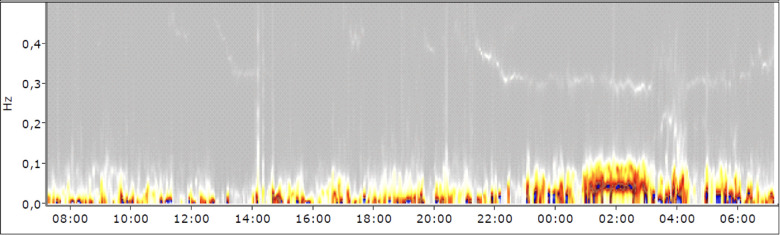
Spectrogram (note: the time on the *X*-axis is the recording time starting from the moment the ECG device was applied).

When assessing the 24 h-ECG recording, a restricted HRV, especially in the range of RMSSD (12.4 ms) and SD1 (8.8 ms), as well as a clear shift in the frequency band (LF/HF) towards the sympathetic nervous system (value above 5) can be seen and should be considered overall as a sign of a disturbed sympathovagal balance (13) (Figures 2, 3).

The spectrogram of the 24 h-ECG recording, especially during daytime hours, clearly shows an increased sympathetic activation with a decrease of the vagus as a sign of tension (Figure 3). Especially in the time between 6:00 PM and 7:30 PM, when the patient kept her patient diary, the sympathetic activity was higher. This can be explained by psychological stress due to that cognitively and emotionally demanding task.

#### Evaluation of HRV during the application phase

**Figure 4:**
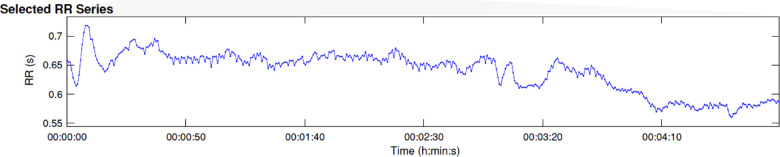
Course of RR intervals during the first 5 min of music application (2.5 min of pain music and 2.5 min of healing music).

For comparability of data, 5-minute intervals are used. Figure 4 shows the course of the RR intervals during the 2.5-minute pain music and the first 2.5 min of the healing music. Strong deflections of the HR are observed in the first 20–30 s of the pain music. Afterwards, the values stabilise at a high level before decreasing about 40 s after the start of the healing music.

**Figure 5:**
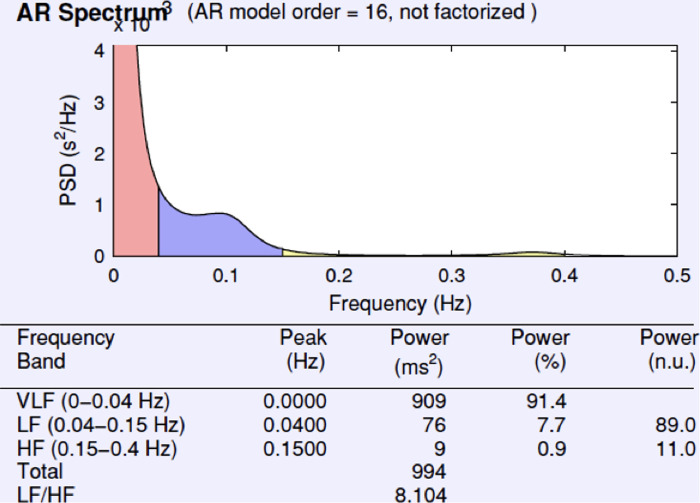
Frequency for RR difference and for RR histogram during the first 5 min of music application.

The frequency-related calculation shows a very pronounced parameter LF/HF of 8.1 during the first 5 min of music application. The vagal activity is clearly subject to sympathetic activity. The relative proportion of HF power is 0.9%, LF power 7.7% and VLF 91.4% (see Figure 5).

## Discussion

The guiding questions for the discussion of the results were mentioned in the introduction.

Unlike a clinical trial, which generates generalisable results, an individual case study offers the possibility to qualitatively reconstruct the inner relationships between the different dimensions. Therefore in the following, the therapy process with its narrative and musical data and the results of the psychometric and cardio-physiological measurements are interrelated and interpreted.

### Psychometric, narrative and music data

Mrs S. was exhausted when she came to the hospital. She had a significantly increased symptom burden as determined by the SCL90 after several years of illness history and various failed treatment attempts. Her biography and verbal statements during the MIPT were in line with her one-sided performance orientation and harshness toward her body, its abilities, sensations, and needs, which manifested in a neglectful attitude towards her own emotional impulses and individual wishes. The data measured by the CTQ reflected her memories of having been mistreated, of beatings, humiliation, and emotional neglect during childhood. The attitudes of her parents who were loyal to the state were regarded by the therapist as formative. These attitudes were based on the social, societal and political norms that once applied in the GDR and were realized in everyday behaviors, including the style of upbringing. In the context of the social climate in a dictatorship with the primacy of the collective, the compulsion to conform to a socialist image of human beings, and the ever-present potential persecution of dissenters, an intertwining of individual and social traumatisation (14) is to be assumed. This interpretation cannot be substantiated on the basis of the material, but it would be an explanation for the expectation of punishment and for the patient's ruthlessness towards herself. A good inner object from whom she received empathy, benevolence, and support was not evident at any point in her narratives.

Interpreting the music composed by Mrs S. to represent her physical pain from a psychodynamic perspective it not only seems mechanical and cruel, but also the way the cello is treated, is reminiscent of abuse. Also the actually soft-sounding marimba was used for creating slamming and relentlessly penetrating noise. In addition, there was the autoaggressive demand during the application phase to increase the dynamics, which drove the therapist into a conflict. On the one hand, she had to follow the guidelines of the method and accurately implement the patient's wishes, and on the other hand, she realised that she was being led to do violence to the patient (musically). The line to re-traumatisation is very thin here. In this case, the therapist relies on the fact that the previous exchange with the patient was stable and that clear stop signs were agreed upon. Nevertheless, the risk remains and represents a considerable demand on therapeutic skills Nevertheless, the patient may have experienced some relief by externalising inner experience, which in turn is the prerequisite for recognising that she herself had also contributed to the tension and fixation of her chronic pain.

It can be seen as success that Mrs S. was able to develop an idea of what pain relief might feel like for her. However, the healing music was associated not only with positive but also with unpleasant memories and was composed in such a way that an exact implementation was fraught with risks due to the intended indeterminacy and irregularity of the sounds. Thus, the potential for failure could be interpreted as the lack of ability to take care of one's own well-being.

From a clinical perspective the patient's fear of physical and psychological relaxation is understandable, because in view of the diagnosed personality disorder it was uncertain whether the existing ego structures would be sufficient to withstand disappointment, anger and grief over the psychological injuries she had suffered. Therefore, the score on the VAS at the end of treatment representing the desired goal has to be discussed carefully. On the one hand, expressing the pain and thus experiencing self-efficacy might have led to feelings of relief. On the other hand, it was impossible to say if her statements corresponded to what Mrs S. actually felt, or if she (unconsciously) wanted to fulfil the expectations of others. For patients with chronic somatoform pain disorder, affect perception, affect differentiation and affect expression can be difficult; this extends to verbal and psychometric verification of what is felt.

For this reason, cardiophysiological measures were included in this case study to obtain primary data. HRV as an indicator of stress and as an vegetative equivalent of affects is not distorted by translation into language, nor by socially desirable behaviors.

### Cardiophysiological data

The 24 h-ECG measurement although still to be considered within the normal range indicated that the patient suffered from permanent tension. The increased HR and sympathetic activity can be observed day and night and peaked when the patient wrote in her diary in the evening after the music therapy. In those moments, she was alone with her thoughts and realisations of how much she had gotten into a vicious cycle due to her autoaggressive behavior exacerbating both pain and panic.

The evaluation of the HRV showed a generally disrupted sympathovagal balance. However, there were also immediate physiological reactions during the application phase of MIPT. There are strong deflections of the HR and the RR distances in the first 20–30 s of the pain music connected to the reported feelings of being punished and remembering her mother's behavior. The RR distances stabilised afterwards but remained at a high level. The strong tension corresponded to the increased pain intensity measured with VAS. This might be related to feeling confronted with her pain and traumatic experiences, with which the patient tried to cope auto-aggressively. From a cardiophysiological point of view, after the beginning of the healing music at 2′30″, no immediate relaxation occurs, but only after about one and a half minutes. This indicates that the patient had difficulty relaxing. She succeeded at least partially, but from a psycho-traumatological point of view, caution was required because, to her, relaxation meant the decline of psychological defence.

The reactualisation of the traumatic experience that occurred during the rest phase and the pain music required stabilisation and self-assurance. The self-composed healing music, the subsequent short conversation, and the surrounding inpatient treatment could also be factors contributing to this. However, it can only be concluded indirectly from subsequent sessions within the framework of MIPT.

### Limitations

This study shows only short-term effects. The long-term impact of MIPT on psychological and physiological well-being cannot be derived from this case report. Also the clinical study in which this patient was participating only gives initial indications of short-term effects (1). However our findings will later help to better explain treatment successes or failures in the context of a larger clinical study (RCT). We recommend an expansion to measurements of intermediate and longer-term impacts of MIPT.

### Future perspective

In the psychotherapeutic treatment of somatoform pain disorders, the perception and verbalisation of feelings are essential to learning to differentiate between body symptoms and affect (15). Therefore, it makes sense to offer arts therapies in multimodal pain treatment, especially music therapy like MIPT. In our concept it was scheduled right at the beginning of the inpatient stay to initiate a creative process that can distract from habitual attitudes and ingrained behaviors and thus promote the achievement of the therapy objective. Physical, sensual-aesthetic, inner- and intrapsychic, and emotional processes are triggered through collaborative music-making (exploring, composing), listening to music and reflecting – aspects which are interrelated on many levels. MIPT can be a suitable starting point and after completion other music therapy methods can also be integrated into the longer-term inpatient and/or outpatient treatments of chronic pain disorders. As shown by the case presented here, a complex set of conditions of a chronic pain disorder becomes visible during few MIPT-sessions, and one should also reckon with a traumatic history. Because MIPT belongs to the confrontational therapy methods, the authors regard the professional experience of the therapist on the one hand and the embedding in a multimodal concept on the other hand as obligatory for its application.

## Data Availability

The original contributions presented in the study are included in the article. Further inquiries can be directed to the corresponding author/s.

## Footnotes

^1^For reasons of style, the course of therapy are written in the present tense.

^2^The ocean drum is a drum, invented by the French composer Olivier Messiaen) that makes sounds similar to those of the ocean.

^3^The sansula made by the HOKEMA company, is a further development of the kalimba, a sound reed instrument from the south of Africa
